# Statistical Study on Additives Used to Improve Mechanical Properties of Polypropylene

**DOI:** 10.3390/polym14010179

**Published:** 2022-01-03

**Authors:** N. S. Yousef

**Affiliations:** Petrochemical Department, Faculty of Engineering, Pharos University, Canal El Mahmoudeya St. Semouha, Alexandria 21311, Egypt; noha.said@pua.edu.eg; Tel.: +20-3-01006741024

**Keywords:** statistical analysis, polypropylene, additives, mechanical properties, T-test, F-test, ANOVA

## Abstract

Polypropylene (PP) is a semi-crystalline polymer that is brittle under severe conditions. To meet industry needs, and to increase the applications of polypropylene, its mechanical properties should be improved. In this research, the mechanical properties of polypropylene, such as tensile strength at break, tensile strength at yield, % elongation, and Young’s modulus, were improved using two types of additives. Additives used were calcium carbonate master batch filler composed of 80% calcium carbonate and 20% polyethylene, and a mixture of linear low-density polyethylene (LLDPE)/low density polyethylene (LDPE). Results showed that both tensile strength at break, and tensile strength at yield, decrease with increasing the amount of both additives. Percentage elongation of PP increased using both additives. The modulus of elasticity of PP increases by increasing the amount of both additives, until a value of 20 wt%. Analysis of variance (ANOVA test) or (F-test) shows significant differences between the effect of different weights of LLDPE/LDPE mixture and calcium carbonate filler on the four mechanical properties of polypropylene studied at a level of 0.05. T-tests are applied to compare between the effect of both calcium carbonate master batch filler and the mixture LLDPE/LDPE on the four mechanical properties of polypropylene studied. T-tests show no significant differences between the effect of both calcium carbonate master batch filler and the mixture LLDPE/LDPE on all mechanical properties of polypropylene studied at a level of 0.05.

## 1. Introduction

Polypropylene is a strong and yet lightweight material with a variety of industrial automotive and everyday uses [1,2]. Polypropylene low specific gravity has enabled it to become portable [2]. Personal computers and hand-held calculators have become possible through the advent of polypropylene and similar plastics. It is an excellent plastic material with a high heat distortion temperature, excellent rigidity, electrical insulation, excellent resistance to folding and ease of molding [3]. Therefore, it is widely used in fiber, daily necessities, packaging films, industrial products, paints and other fields. Among the five common plastics, in terms of production after domestic consumption, polypropylene is ranked second only to polyethylene [4]. Adjusting polypropylene structure and properties will open up new areas of application, and increase the variety and number of new high-performance grades. Additives are vital to enhance mechanical properties of polypropylene. They are introduced to stabilize the polymer, make the polymer easier to process, and enhance its end use properties. To achieve these objectives, many types of additives are required, such as antioxidants, antistatic agents, nucleating agents, light (UV) stabilizers, lubricants, fillers, antimicrobials, slip agents, reinforcing agents, acid scavengers, flame retardants, anti-blocking agents, and colorants [5,6]. Fillers and reinforcements commonly used in polypropylene are calcium carbonate, talc, mica, barite, glass spheres, and carbon and glass fibers [7,8,9,10]. Maximum concentrations of addition are usually 50%, although higher loadings are also used [11,12,13,14]. 

Daniel Eiras and Luiz Antonio Pessan [15] studied the mechanical properties of polypropylene/calcium carbonate nano composites. Four compositions were prepared with a calcium carbonate content of 3 wt%, 5 wt%, 7 wt%, and 10 wt%. The results obtained showed an increase in elastic modulus and yield stress. Abou El-Fettoh A et al. [16] performed a study of some polypropylene nanocomposite properties. Five different compositions of polypropylene/calcium carbonate at a content of 1 wt%, 3 wt%, 5 wt%, 7 wt%, and 10 wt% were prepared using carboxylic acid as compatibilizer. Results showed an improvement in tensile strength at break and elongation at break. Y. W. Leong et al. [17] studied mechanical and thermal properties of talc and calcium carbonate filled polypropylene hybrid composites. Two main types of mineral fillers, calcium carbonate and talc, were added to polypropylene at different filler weight ratios, and were compounded with a twin screw extruder and then injection-molded into dumbbell specimens with an injection-molding machine. Results show an increase in tensile and flexural strength.

A. Buasri et al. [18] studied thermal and mechanical properties of modified CaCO_3_/PP nanocomposites. The results showed that the impact strength of the composite increased by 65%, and the hardness increased by about 5%. Achmad Chafidz et al. [19] studied rheological and mechanical properties of polypropylene/calcium carbonate nanocomposites prepared from masterbatch. The effect of three different nano calcium carbonate compositions (5 wt%, 10 wt%, and 15 wt%) on the rheological/viscoelastic and mechanical properties of polypropylene was investigated. Results showed an improvement in the tensile modulus and toughness (especially at 10 and 15 wt%). On the other hand, the overall flexural and Izod impact of polypropylene properties of the nanocomposites was enhanced.

Mahendra S. B. et al. [20] studied mechanical responses of polypropylene and calcium carbonate nanoparticles. Four compositions of polypropylene and calcium carbonate nanocomposites were prepared in an injection molding machine with varying calcium carbonate percentage (0 wt%, 5 wt%, 10 wt%, 15 wt%). Great increases in tensile breaking strain were obtained with the addition of content of 10% calcium carbonate. Great increases in impact strength with the addition of 5% calcium carbonate. The 15% calcium carbonate has more impact strength than 0%; on the other hand, the 10% calcium carbonate has more impact strength than 15% calcium carbonate. Great increases in flexural strength occurred with the addition of 15% calcium carbonate. 

Blending polypropylene with linear density polyethylene improves stiffness and softening temperature, while producing polypropylene that is easier to process, which extends its applications [21]. 

Ajay Gawali and Lakhan Kalwale [22] studied strength improvement of polypropylene and linear low density polyethylene blend. PP and LLDPE melt blended in proportion of 60:40, 50:50, 80:20 *w*/*w*, respectively. An improvement in tensile strength, elongation at break and impact strength was observed. 

Jia-Horng Lin [23] studied the preparation and compatibility evaluation of polypropylene/high density polyethylene polyblends. Results showed that a 20 wt% of HDPE maintains a certain level of tensile strength and flexural strength, and increases the impact strength of PP/HDPE polyblends by 47%.

The aim of the present work is to apply a statistical study on additives used to improve mechanical properties of polypropylene. The additives used in the work are LLDPE/LDPE mixture, and calcium carbonate master batch filler. Polypropylene mechanical properties studied are tensile strength at break, elongation, modulus of elasticity, and tensile at yield.

## 2. Materials and Methods

### 2.1. Materials

Polypropylene was obtained from Moharam Plastic Company in Abdel Ader (CCI) in Alexandria. Its physical, mechanical, impact, hardness, and thermal properties were measured by the company (CCI), and are shown in Table 1, Table 2 and Table 3, respectively. Calcium carbonate master batch filler composed of 80% CaCO_3_ and 20% linear low-density polyethylene obtained from Egyptian International Plastic Company (EIPC) located in Alexandria City in Egypt. Linear low-density polyethylene (LLDPE) obtained from Egyptian Plastic Industry Company (King Plastic Company) located in Alexandria City in Egypt. Its properties were measured by the company (King Plastic Company), and are shown in Table 4.

Low density polyethylene (LDPE) was obtained from Egyptian European Company (EEC) located in Alexandria City in Egypt. 

Its physical, optical, mechanical, and tensile properties were measured in the company (EEC), and are shown in Table 5, Table 6, Table 7 and Table 8.

### 2.2. Methods

A blank sample of polypropylene was prepared in order to be compared with other samples containing different additive concentrations. Different polypropylene blends containing different additives ratios were prepared with a total weight of 500 g. Additives used were the LLDPE/LDPE mixture, and calcium carbonate master batch filler. Polypropylene pellets and additives were mixed in a mechanical mixer for 8–10 min to provide even distribution, then the mixture was fed to the injection-molding machine to produce the plastic product at a temperature of 250 °C. The samples were left for 24 h to crystallize. After crystallization, the samples were left in an oven to warm and decrease their rigidity. Samples were cut using the cutting machine so that they fit the tensile strength test machine, Charpy unnotched impact tests and the flexural modulus testing machine. Strength, strain and elongation factors were obtained from the curves on the software of the machine and the results were recorded.

### 2.3. Mechanical Properties Measured

Mechanical properties measured were tensile strength at break, tensile strength at yield, % elongation, and Youngs modulus. All previous properties were measured by Sidpec Company in Alexandria Egypt. Each mechanical property test was repeated five times.

### 2.4. Statistical Study on the Effect of Additives on Mechanical Property of Polypropylene

T tests and ANOVA test were done using SPSS software Statistics for Windows, Version 23.0 (IBM SPSS Statistics for Windows, Version 23.0. IBM Corp, Armonk, NY, USA) to study the effect of different additives on four mechanical properties of polypropylene. The effect of both additives, the LLDPE/LDPE mixture and calcium carbonate filler, was studied on the following mechanical properties: tensile strength at break, tensile strength at yield, % elongation, and Youngs modulus. 

## 3. Results and Discussions

### 3.1. Effect of LLDP/LDPE Mixture, and Calcium Carbonate Filler on Mechanical Properties of Polypropylene

#### 3.1.1. Effect of LLDPE/LDPE Mixture, and Calcium Carbonate Filler on Tensile Strength at Break of Polypropylene

**Figure 1:**
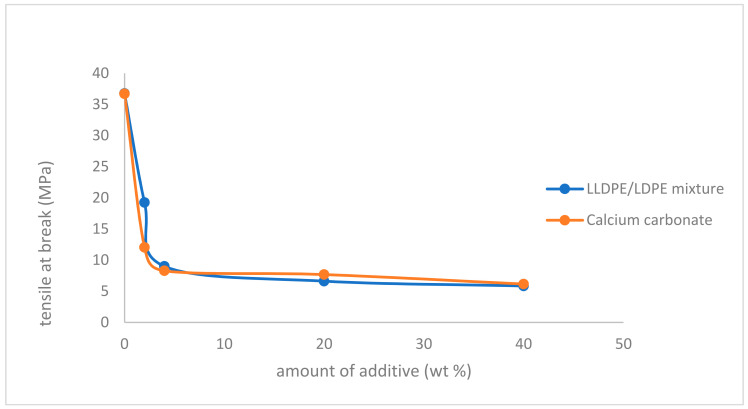
Effect of LLDPE/LDPE mixture, and calcium carbonate filler on tensile strength at break of polypropylene.

A tensile test is the most widely used method to evaluate the mechanical properties of the resultant composites. It is defined as the maximum stress that a material can withstand while being stretched or pulled before failing or breaking, and is measured according to ASTM D 638M and ISO R 527. It is an intensive property; therefore, its value does not depend on the size of the test specimen. However, it is dependent on other factors, such as the preparation of the specimen, the presence of defects, and the temperature of the test environment and material used, such as alloys, composite materials, ceramics, plastics, and wood. Figure 1 shows the effect of both the LLDPE/LDPE mixture and calcium carbonate filler (80% calcium carbonate and 20% polyethylene) on tensile strength at break of polypropylene. As shown in the figure, tensile strength at break decreases with increasing the amount of both additives, which is expected, since the tensile strength at break of polyethylene is lower than that of polypropylene.

#### 3.1.2. Effect of LLDPE/LDPE Mixture, and Calcium Carbonate Filler on % Elongation of Polypropylene

**Figure 2:**
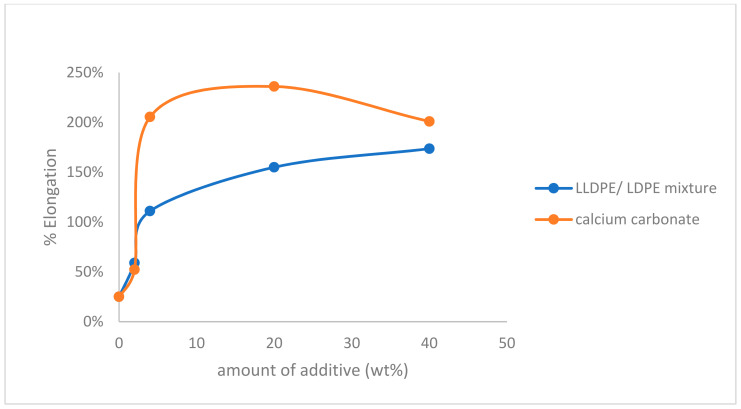
Effect of LLDPE/LDPE mixture, and calcium carbonate filler on % elongation of polypropylene.

Percentage longation of polypropylene was measured according to ASTM D 638M and ISO R 527. As shown in Figure 2, the percentage elongation of the PP increased by increasing the amount of both additives. The increase in the percentage elongation of PP was almost the same using both additives at 2 wt% and 40 wt%. On the other hand, using 4 wt% and 20 wt% of calcium carbonate filler increased percentage elongation much more than that of the LLDPE/LDPE mixture.

#### 3.1.3. Effect of LLDPE/LDPE Mixture, and Calcium Carbonate Filler on Modulus of Elasticity of Polypropylene

**Figure 3:**
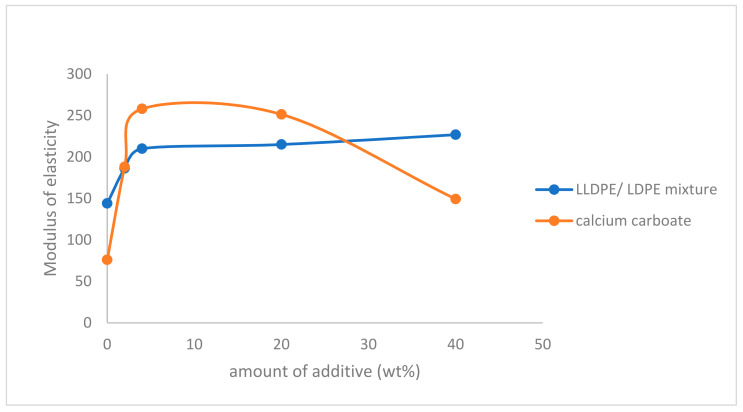
Effect of LLDPE/LDPE mixture, and calcium carbonate filler on modulus of elasticity of polypropylene.

The modulus of elasticity (Young’s modulus) E is a material property that describes its stiffness; it is defined as the ratio of the stress to the strain, is measured according to ASTM D 638M and ISO R 527, and is therefore one of the most important properties of solid materials. As shown in Figure 3, the modulus of elasticity of PP increased by increasing the amount of both additives until a value of 20 wt%. Using more than 20 wt% of the LLDPE/LDPE mixture did not increase the modulus of elasticity too much, On the other hand, more than 20 wt% of calcium carbonate filler decreased the modulus of elasticity of PP. 

#### 3.1.4. Effect of LLDPE/LDPE Mixture, and Calcium Carbonate Filler on Tensile at Yield of Polypropylene

**Figure 4:**
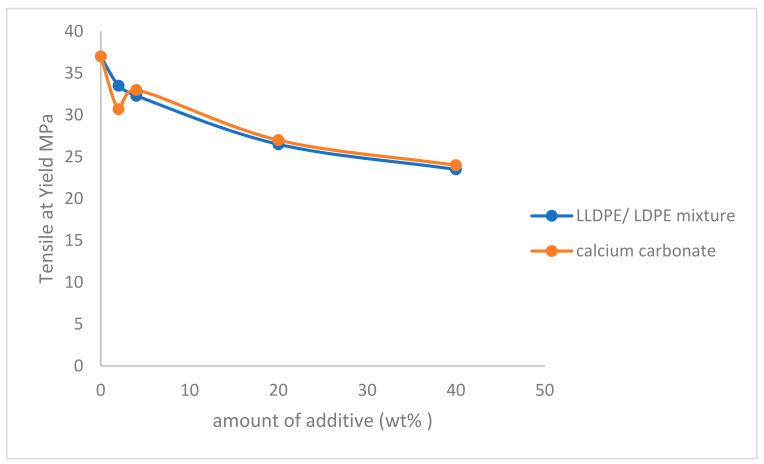
Effect of LLDPE/LDPE mixture, and calcium carbonate filler on tensile at yield of polypropylene.

Tensile at yield is measured according to ASTM D 638M and ISO R 527. Figure 4 shows that tensile strength at yield decreased by increasing the amount of both additives. The percentage decrease in tensile at yield is almost the same by using both additives. It decreases about 35.13% using calcium carbonate filler, and (36.486%) using the LLDPE/LDPE mixture.

### 3.2. F-Test (One Way ANOVA) for the Effect of a Mixture of Linear Low Density Polyethylene/Low Density Polyethylene (1:1) Additive on Mechanical Properties of Polypropylene

One-way analysis of variance (ANOVA) is a statistical method for testing for differences in the means of three or more groups. It is typically used when you have a single independent variable, or factor, and your goal is to investigate if variations, or different levels of that factor, have a measurable effect on a dependent variable. When comparing the means of three or more groups, ANOVA shows if at least one pair of means is significantly different, but without indicating which pair. Moreover, it requires that the dependent variable be normally distributed in each of the groups and that the variability within groups is similar across groups [24].

#### 3.2.1. F-Test (One Way ANOVA) for the Effect of a Mixture of Linear Low Density Polyethylene/Low Density Polyethylene (1:1) Additive on Tensile Strength at Break of Polypropylene

F test shows significant differences between the effect of different weight percentages of the LLDPE/LDPE mixture on tensile strength of polypropylene at a level of 0.05. The additive mixture weight percentages studied were: 0 wt%, 2 wt%, 4 wt%, 6 wt%, 10 wt%, 20 wt%, 30 wt%, and 40 wt%. The test shows the following order of magnitude: 0 wt% > 2 wt% > 4 wt% and 6 wt% > 10 wt% > 20 wt% and 30 wt% > 40 wt%. 

#### 3.2.2. F-Test (One Way ANOVA) for the Effect of a Mixture of Linear Low Density Polyethylene/Low Density Polyethylene (1:1) Additive on Elongation of Polypropylene

Significant differences between the effect of different weight percentages of the LLDPE/LDPE mixture on elongation of polypropylene at a level of 0.05 are shown by F-Test. The additive mixture weight percentages studied were: 0 wt%, 2 wt%, 4 wt%, 6 wt%, 10 wt%, 20 wt%, 30 wt%, and 40 wt%. The test shows the following order of magnitude: 40 wt% > 20 wt% and 30 wt% > 6 wt% and 10 wt% > 4 wt% > 2 wt% > 0 wt%.

#### 3.2.3. F-Test (One Way ANOVA) for the Effect of a Mixture of Linear Low Density Polyethylene/Low Density Polyethylene (1:1) Additive on Modulus of Elasticity of Polypropylene

F test shows significant differences between the effect of different weight percentages of the LLDPE/LDPE additive mixture on the modulus of elasticity of polypropylene at a level of 0.05. The additive mixture weight percentages studied are: 0 wt%, 2 wt%, 4 wt%, 6 wt%, 10 wt%, 20 wt%, 30 wt%, and 40 wt%. The test shows the following order of magnitude: 40 wt% > 6 wt% and 10 wt% and 20 wt% > 30 wt% > 4 wt% > 2 wt% > 0 wt%.

#### 3.2.4. F-Test (One Way ANOVA) for the Effect of a Mixture of Linear Low Density Polyethylene/Low Density Polyethylene (1:1) Additive on Tensile at Yield of Polypropylene

Significant differences between the effect of different weight percentages of LLDPE/LDPE mixture on tensile at yield of polypropylene at a level of 0.05 were shown by F-Test. The additive mixture weight percentages studied are: 0 wt%, 2 wt%, 4 wt%, 6 wt%, 10 wt%, 20 wt%, 30 wt%, and 40 wt%. The test shows the following order of magnitude: 0 wt% > 2 wt% > 4 wt% > 6 wt% > 10 wt% > 20 wt% and 30 wt% and 40 wt%.

#### 3.2.5. F-Test (One Way ANOVA) for the Effect of Calcium Carbonate Master Batch Filler Additive on Tensile Strength of Polypropylene

Significant differences between the effect of different weight percentages of calcium carbonate master batch filler additive on tensile at yield of polypropylene at a level of 0.05 were shown by F-Test. The filler weight percentages studied are: 0 wt%, 1 wt%, 2 wt%, 3 wt%, 4 wt%, 20 wt%, and 40 wt%. The test shows the following order of magnitude: 0 wt% > 1 wt% > 2 wt% > 3 wt% and 4 wt% and 20 wt% > 40 wt%.

#### 3.2.6. F-Test (One Way ANOVA) for the Effect of Calcium Carbonate Master Batch Filler Additive on Elongation of Polypropylene

F test shows significant differences between the effect of different weight percentages of calcium carbonate master batch filler additive on elongation of polypropylene at a level of 0.05. The filler weight percentages studied were: 0 wt%, 1 wt%, 2 wt%, 3 wt%, 4 wt%, 20 wt%, and 40 wt%. The test shows the following order of magnitude: 4 wt% and 20 wt% and 40 wt% > 3 wt% > 1 wt% and 2 wt% > 0 wt%.

#### 3.2.7. F-Test (One Way ANOVA) for the Effect of Calcium Carbonate Master Batch Filler Additive on Modulus of Elasticity of Polypropylene

F test shows significant differences between the effect of different weight percentages of calcium carbonate master batch filler on the modulus of elasticity of polypropylene at a level of 0.05. The filler weight percentages studied were: 0 wt%, 1 wt%, 2 wt%, 3 wt%, 4 wt%, 20 wt%, and 40 wt%. The test shows the following order of magnitude: 3 wt% and 4 wt% and 20 wt% > 2 wt% and 40 wt% > 0 wt% > 1 wt%. 

#### 3.2.8. F-Test (One Way ANOVA) for the Effect of Calcium Carbonate Master Batch Filler Additive on Tensile at Yield of Polypropylene

Significant differences between the effect of different weight percentages of calcium carbonate master batch filler on tensile at yield of polypropylene at a level of 0.05 were shown by F-Test. The weight percentages studied were: 0 wt%, 1 wt%, 2 wt%, 3 wt%, 4 wt%, 20 wt%, and 40 wt%. The test shows the following order of magnitude: 0 wt% > 1 wt% and 2 wt% and 3 wt% and 4 wt% > 20 wt% and 40 wt%.

### 3.3. T-Tests

T tests were applied to compare between the effect of both calcium carbonate master batch filler and the LLDPE/LDPE mixture on the four mechanical properties studied. 

#### 3.3.1. T-Test on the Effect of Both Calcium Carbonate Master Batch Filler and the Mixture LLDPE/LDPE on Tensile Strength of Polypropylene

The *t* test showed that there were no significant differences between the effect of both calcium carbonate master batch filler and the LLDPE/LDPE mixture on tensile strength of polypropylene at a level of 0.05, nor for weight percentages of 2 wt%, 4 wt%, 20 wt%, and 40 wt%.

#### 3.3.2. T-Test on the Effect of Both Calcium Carbonate Master Batch Filler and the Mixture LLDPE/LDPE on Elongation of Polypropylene

No significant differences between the effect of both calcium carbonate master batch filler and the LLDPE/LDPE mixture on elongation of polypropylene were shown by t test at a level of 0.05, nor for weight percentages of 2 wt%, 4 wt%, 20 wt%, and 40 wt%.

#### 3.3.3. T-Test on the Effect of Both Calcium Carbonate Master Batch Filler and the Mixture LLDPE/LDPE on Modulus of Elasticity of Polypropylene

T test shows that there were no significant differences between the effect of both calcium carbonate master batch filler and the LLDPE/LDPE mixture on the modulus of elasticity of polypropylene at a level of 0.05, nor for weight percentages of 2 wt%, 4 wt%, 20 wt%, and 40 wt%.

#### 3.3.4. T-Test on the Effect of Both Calcium Carbonate Master Batch Filler and the Mixture LLDPE/LDPE on Tensile at Yield of Polypropylene

No significant differences between the effect of both calcium carbonate master batch filler and the LLDPE/LDPE mixture on tensile at yield of polypropylene were shown by t test at a level of 0.05, nor for weight percentages of 2 wt%,4 wt%, 20 wt%, and 40 wt%. 

## 4. Conclusions

Mechanical properties of polypropylene were improved when calcium carbonate master batch filler, and a mixture of LLDPE/LDPE, were added to it. Results showed that both tensile strength at break, and tensile strength at yield, decreased with increasing the amount of both additives, which is expected, since the tensile strength at break of polyethylene is lower than that of polypropylene. Percentage elongation of PP increased using both additives, almost the same using both additives at an amount of 2 wt% and 40 wt%. On the other hand, using 2 wt% and 20 wt% of calcium carbonate additive increased percentage elongation much more than that of the LLDPE/LDPE mixture. The modulus of elasticity of PP increases by increasing the amount of both additives, until a value of 20 wt%. Using more than 20 wt% of LLDPE/LDPE mixture did not increase the modulus of elasticity too much, On the other hand, more than 20 wt% of calcium carbonate additive decreased the modulus of elasticity of PP. F test (one way ANOVA) showed significant differences between the effect of different weights of the LLDPE/LDPE mixture and calcium carbonate additives on all mechanical properties studied at a level of 0.05. T tests showed no significant differences between the effect of both calcium carbonate master batch filler and the mixture LLDPE/LDPE on all mechanical properties studied at a level of 0.05.

## Figures and Tables

**Table 1:** Physical Properties of polypropylene.

Physical Properties	Method	Results
Density (Method D)	ISO 1183	0.90 g/cm^3^
Melt Flow Rate (MFR) (230 Degrees Celsius/2.16 Kg)	ISO 1133	25 g/10 min

**Table 2:** Mechanical Properties of polypropylene.

Mechanical Properties	Method	Results
Tensile Stress at Yield	ISO 527-1, 2	32 MPa
Flexural Modulus	ISO 178	1350 Mpa
Shore Hardness (Shore D)	ISO 868	70

**Table 3:** Impact Properties of polypropylene.

Tests	Method	Results
Charpy Un-notched Impact Strength (23 Degrees Celsius, Type 1, Edgewise) (0 Degree Celsius, Type 1, Edgewise)	ISO 179	105 kJ/m^2^ 25 kJ/m^2^
Charpy Notched Impact Strength (23 Degrees Celsius, Type 1, Edgewise, Notch A)	ISO 179	2 kJ/m^2^

**Table 4:** Properties of Linear Low Density poly ethylene.

Property	Unit	Test Method	Typical Value
Melt Index	g/10 min	D1238	4
Density	g/cm^3^	D1505	0.932
Flexural Modulus	MPa	D790	1350
unnotched Izod Impact at 23 degree Celsius	J/m	D256/A	NB
E.S.C. R	h	D1693	>1000

**Table 5:** Physical Properties of Low Density Poly ethylene.

Physical Properties	Unit	Results
Melt Flow Rate	dg/min	0.3
Density	Kg/m^3^	921

**Table 6:** Optical Properties of Low Density Poly ethylene.

Optical Properties		Test	Results
Gloss (45)	%	ASTM D 2457	46
Haze	%	ASTM D 1003A	12
Clarity	mV	SABTEC method	65

**Table 7:** Mechanical Properties of Low Density poly ethylene.

Mechanical Properties	Units	Test	Results
Impact Strength	kJ/m	ASTM 4272	35
Tear Strength TD	kN/m	-	25
Tear Strength MD	kN/m	-	20

**Table 8:** Tensile Test Properties of Low Density poly ethylene.

Properties	Units		Results
Yield Stress TD	MPa	-	11
Yield Stress MD	MPa	-	12
Tensile Stress at Break TD	Mpa	-	26
Tensile Stress at Break MD	MPa	-	29
Strain Break TD	%	-	>500
Strain Break MD	%	-	>200
Modulus of Elasticity TD	MPa	-	190
Modulus of Elasticity MD	MPa	-	180
Coefficient of Friction		ASTM D 1894	0.7
Blocking	g	-	<5
Re-Blocking	g	-	30
